# The Science and Art of Surgery; Being a Treatise on Injuries, Diseases, and Operations

**Published:** 1859-05

**Authors:** 


ART. VI.—*The Science and Art of Surgery; being a Treatise on Injuries, Diseases, and Operations.* By JOHN ERICHSEN, Professor of Surgery, and of Clinical Surgery in University College, etc. Second American, from the second enlarged and carefully revised London edition. 8vo., pp. 996. Philadelphia: Blanchard & Lea, 1859.

THE appearance of another edition of this deservedly popular and standard work, enlarged as it is by nearly one hundred pages of new matter and a large number of wood-cuts, will be welcomed by the profession of this country, which received Mr. Erichsen five years ago as an entire stranger. The present edition has been carefully revised, and many valuable additions have been made. As a complete system of surgery, however, it cannot be considered as perfect, as some subjects have been neglected which should have received attention. We specify, in particular, the diseases of the eye and ear, which have been entirely excluded from its pages. The faults of omission, however, can be easily remedied, and do not detract much from the value of the work, which now holds the same position as that of Watson on the Practice of Medicine. We have no hesitation in saying that Mr. Erichsen has succeeded in producing the best surgical work in the English language, and as such we give it our hearty commendation. We notice that the present edition appears without the name of an American editor, but a few notes and wood-cuts have been added to illustrate American surgery.

